# Vaccination Officers and Their Duties

**Published:** 1899-11-18

**Authors:** 


### VACCINATION OFFICERS AND THEIR DUTIES.

The difficulty about the duties of the vaccination officer which has led to so much correspondence in the daily press under the above heading, and as a side issue has served as an excuse for the rebellious attitude taken up by the Leicester Guardians, seems to have arisen from an unfortunate error on the part of the Local Government Board. The facts are simple enough. In the Vaccination Act, 1871, it was made obligatory that all Boards of Guardians "shall appoint and pay one or more such officers (in this Act referred to as vaccination officers)," to "prosecute persons charged with offences against that Act [*namely, the principal Act of* 1867] or otherwise to enforce its provisions." According to this, when the Guardians had appointed and had paid their officer they had done with him, and all they had to do with him further was to take care that he did his work. It soon seems to have been thought, however, that the vaccination officer, being the servant of the guardians, ought to be to some extent under their control, and the Vaccination Act, 1874, having given to the Local Government Board the power to make rules, orders, and regulations, with respect to the proceedings to be taken by the guardians or their officers for the enforcement of the provisions of the Vaccination Acts, the Board straightway issued an Order which, while limiting the power of the vaccination officer in regard to the conduct of legal proceedings "until he shall have brought the circumstances of the case under the notice of the Guardians and received their special directions thereon," at the same time required the Guardians to "give directions authorising the vaccination officer to institute and conduct" such proceedings. Now there can hardly be a doubt that the action of the Local Government Board in issuing this Order was the starting point of a muddle, for while the Act was plainly in favour of the independence of the vaccination officer, the Order as plainly threw the responsibility on the Guardians. Then came a legal decision, according to which it was the Act and not the Order which was to be maintained. Under these circumstances it would undoubtedly have been a statesmanlike proceeding to have taken the opportunity of the introduction of a new Vaccination Bill to have got this difficulty made clear. The promoters of the Bill thought otherwise, and on the principle, we suppose, of letting sleeping dogs lie, they did not raise this thorny question, but treated it as solved by the decision of the law courts. Other people, however, chose to take it as solved by the Order of the Local Government Board, and hence the muddle. This is but another example of the manner in which difficulties were shirked in the passing of that unfortunate Act.

